# Serum Proteome Alterations in Patients with Cognitive Impairment after Traumatic Brain Injury Revealed by iTRAQ-Based Quantitative Proteomics

**DOI:** 10.1155/2017/8572509

**Published:** 2017-01-30

**Authors:** Xin-gui Xiong, Qinghua Liang, Chunhu Zhang, Yang Wang, Wei Huang, Weijun Peng, Zhe Wang, Zi-an Xia

**Affiliations:** ^1^Institute of Integrated Medicine, Xiangya Hospital, Central South University, Changsha, Hunan 410008, China; ^2^Department of Integrated Traditional Chinese & Western Medicine, The Second Xiangya Hospital, Central South University, Changsha, Hunan 410011, China

## Abstract

*Background*. Cognitive impairment is the leading cause of traumatic brain injury- (TBI-) related disability; however, the underlying pathogenesis of this dysfunction is not completely understood.* Methods*. Using an isobaric tagging for relative and absolute quantitation- (iTRAQ-) based quantitative proteomic approach, serum samples from healthy control subjects, TBI patients with cognitive impairment, and TBI patients without cognitive impairment were analysed to identify differentially expressed proteins (DEPs) related to post-TBI cognitive impairment. In addition, DEPs were further analysed using bioinformatic platforms and validated using enzyme-linked immunosorbent assays (ELISA).* Results*. A total of 56 DEPs were identified that were specifically related to TBI-induced cognitive impairment. Bioinformatic analysis revealed that a wide variety of cellular and metabolic processes and some signaling pathways were involved in the pathophysiology of cognitive deficits following TBI. Five randomly selected DEPs were validated using ELISA in an additional 105 cases, and the results also supported the experimental findings.* Conclusions*. Despite limitations, our findings will facilitate further studies of the pathological mechanisms underlying TBI-induced cognitive impairment and provide new methods for the research and development of neuroprotective agents. However, further investigation on a large cohort is warranted.

## 1. Introduction

Traumatic brain injury (TBI) is a major public health concern that affects 12% of the general population and results in high rates of death and disability worldwide [1]. Approximately 65% of moderate-to-severe TBI patients suffer from long-lasting cognitive deficits, including deficits in memory capacity, attention, executive functions, and general cognitive intelligence [2, 3]. These deficits impose a personal and economic burden that is difficult to quantify. Despite significant efforts, the pathologies underlying TBI-induced cognitive impairment are poorly understood, and effective disease-modifying treatment strategies are lacking [4, 5]. Thus, investigation of alternative disease mechanisms of TBI-induced impairment are required to better understand its pathophysiology and to yield effective therapeutic agents [4].

Due to the complexity and heterogeneity of TBI-induced cognitive impairment, it is likely that multiple candidate proteins present in networks are perturbed, leading to the spectrum of cognitive symptoms. Currently, with the advent of quantitative proteomic technologies using an isobaric labelling strategy, it has become possible to quantify several proteins in a single experiment for the comparative study of global protein regulation across various biological samples, and this method has been widely applied to elucidate disease mechanisms [6–9]. Therefore, this method represents an exciting new approach that can help to address the complex pathology of TBI-induced cognitive impairment. A novel quantitative proteomic technology, isobaric tagging for relative and absolute quantitation (iTRAQ), has recently become a powerful tool to characterize protein expression during different physiological and pathological states. In particular, several studies have successfully applied this novel quantitative proteomic technology to identify the biomarkers for TBI in rats with diffuse axonal injury [10], patients with elevated intercranial pressure [11], and an ApoE mouse model [12].

However, none of the previous studies applied a quantitative proteomic technology to identify global protein changes and pathways perturbed in post-TBI cognitive impairment using clinical samples. In this study, an iTRAQ-based quantitative proteomics approach was adopted to identify and quantity the differentially expressed proteins (DEPs) in serum samples from TBI patients with cognitive impairment. In addition, DEPs were further analysed by bioinformatic platforms and validated by enzyme-linked immunosorbent assays (ELISA). These findings will further the understanding of the pathophysiological mechanisms underlying post-TBI cognitive impairments.

## 2. Material and Methods

### 2.1. Ethics Statement

All protocols involving the use of human subjects were reviewed and approved by the Ethics Committee of Central South University, Changsha, China (Grant no. 201404366), and all experiments were performed in accordance with the Declaration of Helsinki. Written informed consent was obtained from all participants (or their legal guardians) enrolled in this study.

### 2.2. Study Subjects

All participants presented at the Brain Trauma Specialist Department, Department of Encephalopathy of the National Key Specialty, and the Health Centre of the Xiangya Hospital, Central South University, Changsha, China, between February 2014 and December 2014. The subjects were divided into three groups: healthy controls (HC group), TBI patients without cognitive deficits (negative group), and TBI patients with cognitive deficits (positive group).

As described in our previous study [13], TBI patients were screened to meet the following inclusion criteria: (i) age > 18 and <65 years; (ii) a history of moderate-to-severe TBI (defined as an initial Glasgow Coma Scale (GCS) score of 12 or less); (iii) 1 to 6 months after initial injury; (iv) being without various types of extracranial injuries; and (v) no anticipated/pending neurosurgical operative intervention.

Individuals were excluded if they met any of the following criteria: (i) serious conditions causing mental disability prior to the TBI, such as a developmental handicap (Down's syndrome), residual disability after previous TBI, confirmed dementia, or serious chronic mental illness (schizophrenia, psychosis, or well-confirmed bipolar disorder); (ii) severe renal or hepatic impairment; (iii) uncontrolled cardiovascular disease; (iv) a current history of severe abuse of drugs or alcohol; and (v) being pregnant or lactating.

When TBI patients were recruited into study, the overall level of cognitive and behavioural functioning of them was assessed using the Rancho Los Amigos Scale (RLAS, also referred to as “Rancho” or the “Levels of Cognitive Functioning Scale”) by two doctors [14, 15]. TBI patients who obtained scores ranging from Level 1 to Level 8 were classified into the positive group, and those with a level of cognitive and behavioural functioning higher than Level 8 were classified into the negative group.

In addition, the healthy control group was composed of healthy volunteers with no current or previous lifetime history of neurological diseases or systemic medical illness. Healthy controls were matched with TBI patients for age and gender. The demographic and clinical chemistry characteristics of enrolled subjects are shown in Table 1.

### 2.3. Sample Collection and Preparation

The subjects fasted for at least 12 hours before blood was drawn. The blood samples were obtained specifically for the purpose of this study and were coded to maintain anonymity. Relevant medical data were recorded and coded to match the extracted blood samples. A 3 mL blood serum sample was collected from each enrolled subject. The serum samples were placed in Eppendorf tubes without anticoagulant at 4°C and allowed to stand for 1 h. The sample was centrifuged at 3000*g* at 4°C for 15 min. The supernatant consisted of the total serum proteins. Then, the sample was divided into 0.5 mL aliquots and stored at −80°C in a refrigerator for future use.

Pooled serum samples were generated by combining equal volumes of the 15 individual plasma samples from each group (*n* = 16). The high-abundance proteins of each serum pool were depleted using ProteoMiner Protein Enrichment Kits (Bio-Rad, USA) according to the manufacturer's instructions. The protein concentration was determined using a Bradford Protein Assay Kit (Amesco, Ohio, USA) [16].

### 2.4. iTRAQ-Based Quantitative Proteomics Analysis

As described in our previous study [17], the analytic processes were performed by the Beijing Protein Innovation Co., Ltd., Beijing, China, and included protein digestion, iTRAQ labelling, strong cation exchange (SCX) fractionation, LC-MS/MS analysis, protein identification, and protein quantitation.

Briefly, trypsin digestion and iTRAQ labelling were performed according to the manufacturer's protocol (Applied Biosystems). First, 50 *μ*g of protein from each pooled sample was reduced, alkylated, and digested overnight at 37°C with trypsin. Then, according to the iTRAQ Reagent Multiplex Kit (Applied Biosystems) protocol, the tryptic peptide solution of each sample was labelled with iTRAQ reagents as follows: control group, iTRAQ reagent 113; negative group, iTRAQ reagent 114; positive group, iTRAQ reagent 115. The tryptic peptide samples were labelled, mixed, and dried before further analyses.

The mixed peptides were fractionated by SCX chromatography on an ultimate high-performance liquid chromatography (HPLC) system (Shimadzu, Kyoto, Japan) with an SCX column (Luna SCX 100A, Phenomenex). Based on the SCX chromatograms, 10 SCX fractions were collected along the gradient. Each SCX fraction was dried, dissolved, and then analysed on a reverse-phase liquid chromatography column (Strata-X C18 column, 5 *μ*m, 300 A, 100 mm × 75 mm, Phenomenex).

Mass spectrometry (MS) analysis of the iTRAQ-labelled samples was performed on a Q Exactive LC-MS/MS (ThermoFisher Scientific, Waltham, MA, USA) mass spectrometer. Sequences for the peptide and reporter ions were generated to identify the protein from which the peptide originated. To minimize the effect of experimental variation, three independent MS/MS runs were performed for each sample.

Proteome Discoverer Software (Thermo Scientific version 1.3) was used for the data acquisition and quantification. The data sifted by Proteome Discoverer were used to identify proteins using Mascot (version 2.3.0, Matrix Science, London, UK) software and the Uniprot-rat database (http://www.uniprot.org/). The quantitative result of the peptide was the ratio of the signal intensity value of the reference sample (normal sample) label to the signal strength values of other labels. The protein quantitative ratio was calculated as the median of all peptide ratios. The final quantitative result was normalized to the median ratio of each label. The fold change of the DEPs was calculated as the average value from the protein iTRAQ ratios. The DEPs were identified according to the following criteria: the minimum fold change was ±1.2, the difference was statistically significant (*P* < 0.05), and no less than one peptide within the 95% confidence interval was included [18].

### 2.5. Bioinformatic Analysis

As described in a previous study [19], a list containing all DEPs identified previously was submitted to the Gene Ontology Consortium, and PANTHER database analysis tools were applied for functional annotation and enrichment analysis [20]. Pathway analysis was performed using the KEGG database. Predicted protein-protein interactions (PPIs) were generated and visualized using STRING software [21]. *P* values and Benjamini-corrected *P* values less than 0.05 were considered significant.

### 2.6. ELISA Validation

As described in our previous study [22], the serum levels of selected DEPs were measured using an ELISA quantitation kit (USCN Life Sciences, Wuhan, China) following protocols recommended by the manufacturer.

### 2.7. Statistical Analyses

All data are expressed as the means ± SE. One-way ANOVA was used to compare the differences between the groups. All statistical analyses were conducted using the SPSS (version 22.0, Chicago, IL). *P* values < 0.05 were considered to indicate statistically significant differences.

## 3. Results

### 3.1. Identification and Relative Quantification of Dysregulated Proteins

**Figure 1:**
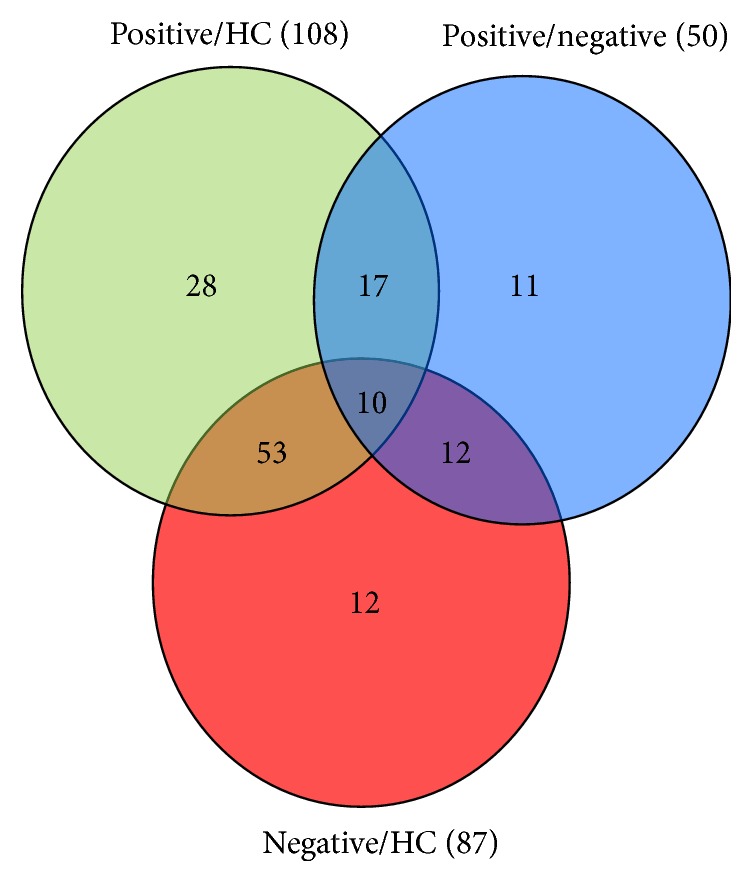
Venn diagram showing the number of differentially expressed proteins (DEPs) and their overlap. The results indicated 108 proteins showed differential expression in the positive versus healthy control (HC) groups (green cycle), 50 proteins in the positive versus negative groups (blue cycle), and 87 proteins in the negative versus HC groups (red cycle). A total of 56 DEPs which included 28 DEPs in the positive versus negative comparison, 11 DEPs in the positive versus negative comparison, and 17 DEPs in the overlapping regions between both comparisons (positive versus negative and positive versus negative) were specific to the positive group.

Using iTRAQ-based quantitative proteomics, a total of 331,259 MS/MS spectra were obtained, of which 40,273 were matched. Then, 48,350 PSMs were assigned to 3079 peptides after 1% FDR was applied. Through this strategy, 359 proteins were identified for further study. Of the 359 proteins, we identified 50 DEPs in the positive and negative subjects, including 23 upregulated and 27 downregulated proteins. Meanwhile, 108 DEPs were identified in the positive and control subjects, including 54 upregulated and 54 downregulated proteins. Additionally, 87 DEPs were identified in the negative and control subjects, including 37 upregulated and 40 downregulated proteins. As shown in Figure 1, of these dysregulated proteins, 56 DEPs were specific to TBI-induced cognitive impairment (Figure 1, Table 2).

### 3.2. Functional Classification of DEPs

**Figure 2:**
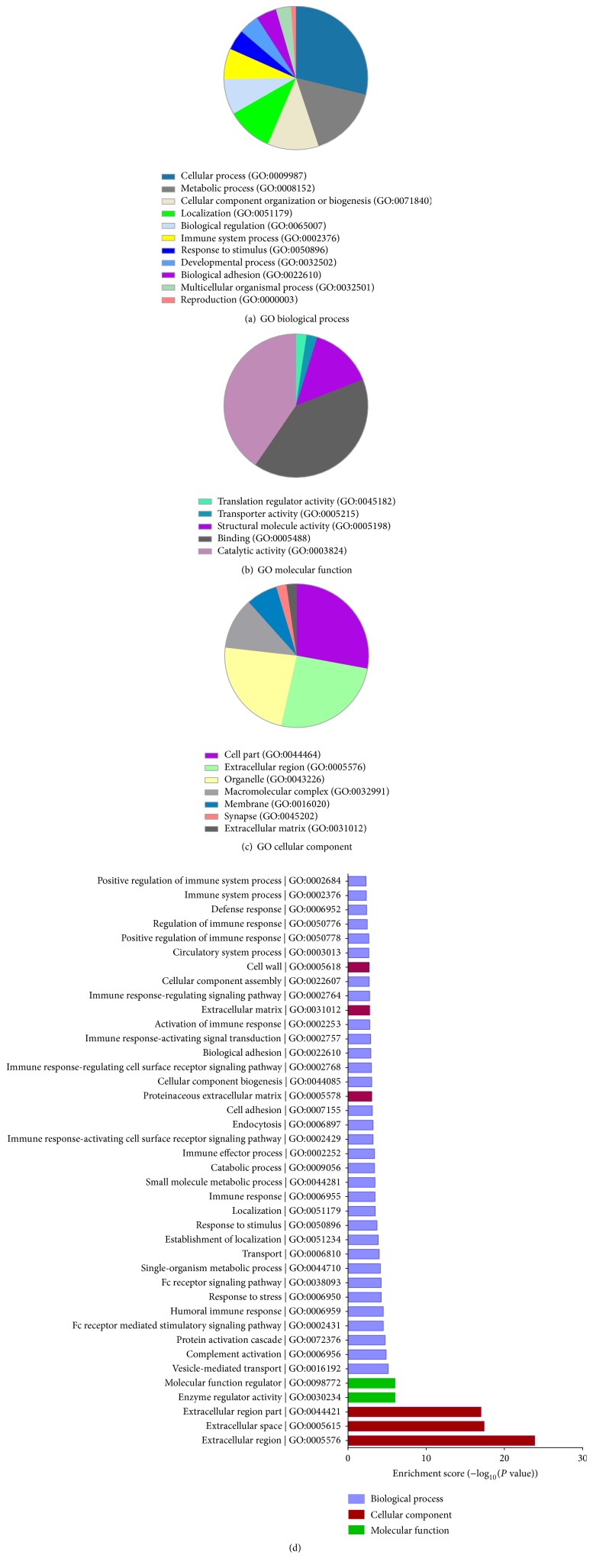
GO analysis of the differentially expressed proteins (DEPs). All identified proteins were functionally annotated in GO database according to their biological process (a), molecular function (b), and cellular component (c). In addition, the GO term enrichment analysis was conducted, and the significantly enriched categories (*P* < 0.05) were recorded (d).

To gain insight into the biological changes in TBI patients with cognitive impairment, the DEPs were categorized according to the following Gene Ontology (GO) classes: biological process, molecular function, and cellular components (Figure 2). In the biological processes class, most of the DEPs were mainly associated with cellular component organization or biogenesis (GO:0071840, 11.5%), cellular processes (GO:0009987, 28.7%), localization (GO:0051179, 10.3%), and metabolic processes (GO:0008152, 16.1%) (Figure 2(a)). In the molecular function group, the DEPs were mainly found to be involved in binding (GO:0005488, 40.5%), structural molecule activity (GO:0005198, 14.3%), and catalytic activity (GO:0003824, 40.5%) (Figure 2(b)). In the cellular components class, the DEPs were mainly located in macromolecular complexes (GO:0032991, 11.6%), the cellular region (GO:0044464, 27.9%), organelles (GO:0043226, 23.3%), and the extracellular region (GO:0005576, 25.6%) (Figure 2(c)).

To determine whether the DEPs were enriched in certain groups, we employed the PANTHER Overrepresentation Test and used the Bonferroni correction for multiple comparisons. As shown in Figure 2(d), we found that the DEPs involved in vesicle-mediated transport (GO:0016192) were the most significantly enriched group in the biological process group (*P* = 0.000007), the DEPs involved in enzyme regulatory activity (GO:0030234) were the most significantly enriched group in the molecular function group (*P* = 0.000001), and the DEPs involved in the extracellular region (GO:0005576) were the most significantly enriched group in the cellular component group (*P* < 0.00001).

### 3.3. KEGG Pathway Analysis

Because we were interested in the signaling pathways enriched in DEPs, a KEGG pathway analysis was performed. As shown in Table 3, 16 significantly enriched pathways were found, including pathways involved in Alzheimer's disease (AD) (ko05010, *P* = 0.015594). Additionally, glyceraldehyde-3-phosphate dehydrogenase (GAPDH), calmodulin (CaM), and lipoprotein lipase (LPL) were involved in the AD pathways (Figure S1 in Supplementary Material available online at https://doi.org/10.1155/2017/8572509).

### 3.4. Protein-Protein Interaction (PPI) Analysis of the DEPs

**Figure 3:**
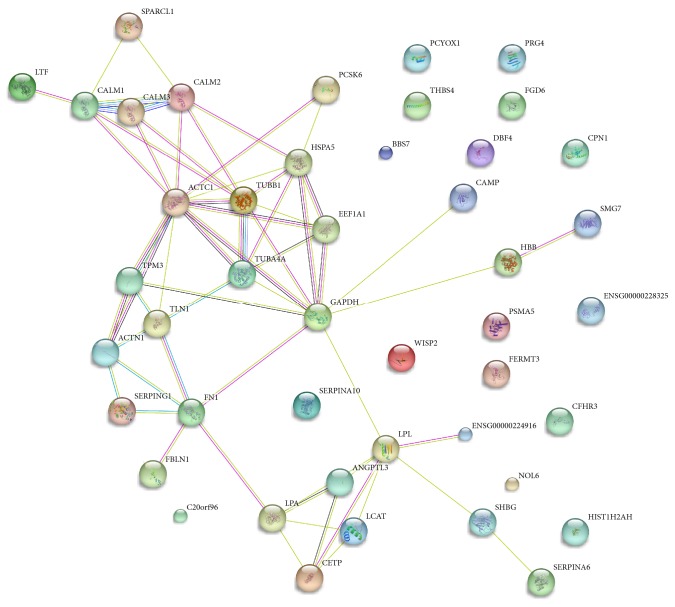
Protein-protein interactions for the differentially expressed proteins identified using iTRAQ-based proteomics were analysed with STRING V10.0. In the network, the proteins are represented as nodes. The colors of the lines connecting the nodes represent different evidence types for the protein linkage.

The 56 DEPs identified in the current study were submitted to STRING to assess the PPI networks (Figure 3). Of the 56 identified DEPs, 45 were mapped in PPI networks, 29 were interconnected, and 16 proteins did not show any type of connection at the selected confidence level (STRING score = 0.4). In addition, we found that CaM (CALM1, CALM2, and CALM3), LPL, GAPDH, tubulin alpha-4A (TUBA4A), and actin alpha cardiac muscle 1 (ACTC1) were located in a network hub and exhibited a complex relationship with the other proteins.

### 3.5. Verification of the DEPs Using ELISA

Based on the results of the bioinformatic analysis and the correlations with disease pathogenesis, five candidate DEPs, namely, GAPDH, CaM, apolipoprotein(a) (APO(a)), thrombospondin-4 (THBS4), and Talin-1 (TLN1), were selected for validation in an additional 105 cases using ELISA. These cases included 35 TBI patients with cognitive impairment, 35 TBI patients without cognitive impairment, and 35 healthy controls.

**Figure 4:**
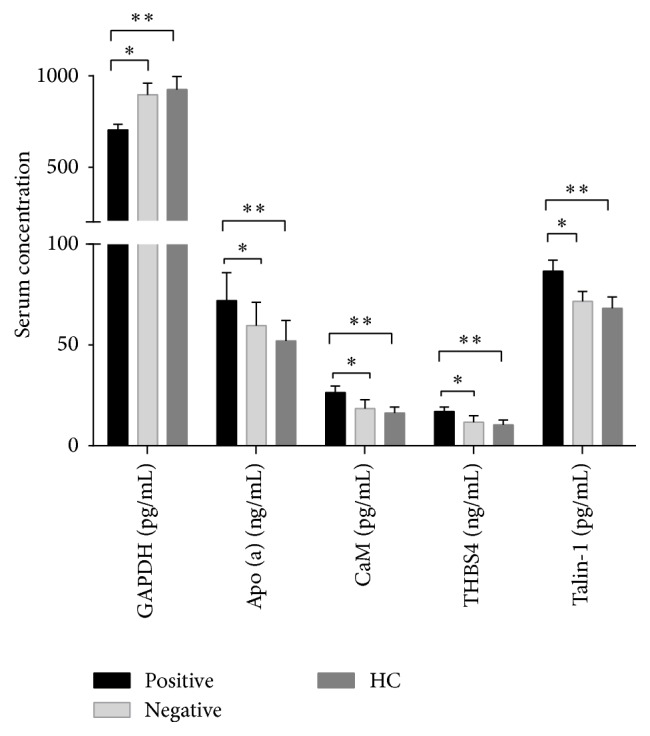
Serum proteins levels among the positive, negative, and HC group. A *P* value less than 0.05 indicates statistical significance using the Mann–Whitney *U*-test. ^*∗*^*P* < 0.05 and ^*∗∗*^*P* < 0.01.

As shown in Figure 4, consistent with the data obtained in the proteomic studies, the results revealed significantly increased serum CaM, APO(a), THBS4, and Talin-1 levels (*P* = 0.007, *P* = 0.018, *P* = 0.0019, and *P* = 0.006, resp.) and significantly reduced GAPDH levels (*P* = 0.025) in the positive group compared to the negative group. In addition, significant differences in GAPDH, CaM, APO(a), THBS4, and Talin-1 levels were also observed between the positive and control groups (*P* = 0.007, *P* < 0.005, *P* = 0.001, *P* = 0.006, and *P* = 0.001, resp.).

## 4. Discussion

In our study, using an iTRAQ-based quantitative proteomic approach, a total of 56 DEPs were found which displayed quantitative changes unique to TBI patients with cognitive deficits relative to healthy controls and TBI patients without cognitive deficits. Of these DEPs, 30 were downregulated, including LPL and GAPDH, and 26 were upregulated, including APO(a), THBS4, and CaM.

According to the bioinformatic analysis, the 56 DEPs were suggested to be involved in a wide variety of cellular and metabolic processes, including immunity and inflammation, transportation of important regulatory biomolecules, blood coagulation, and other cell processes, and a sizeable group of significantly differentiated pathways with important biological functions. Moreover, the results of PPI analysis indicated that TBI-induced cognitive impairment is a multifactorial process of pathological progress that involves various proteins that interact with each other including the SPARCL1-CALM-ACTC1-TUBA4A-GAPDH-LPL-SHBG-SERPINA6 network. In addition, the ELISA validation results confirmed the proteomics analysis findings to some extent.

Interestingly, our results revealed that AD signaling pathways (including LPL, GAPDH, and CaM) might play an important role in the pathophysiology of post-TBI cognitive impairments. Numerous epidemiological studies have indicated that TBI can increase the risk of developing AD, which is the most common form of dementia [23, 24]. Our present findings provide further evidence of the association between TBI and AD from the perspective of serum protein expression profiling.

As a classical glycolytic enzyme, GAPDH was validated by ELISA as being significantly downregulated in the TBI patients with cognitive impairment. In addition, GAPDH has been suggested to have high affinity for AD-associated proteins, including *β*-amyloid, *β*-amyloid precursor protein, and tau [25], and to be involved in the NO/GAPDH/Siah-1 apoptotic cell death cascade [26], particularly in neuronal cell death associated with neurodegenerative diseases [25, 27]. CaM, the major intracellular Ca^2+^ ion-binding protein, was significantly upregulated in the TBI patients with cognitive impairment. As a primary Ca^2+^ signal transducer, CaM responds to cytosolic Ca^2+^ fluxes by binding to and regulating the activity of target CaM-binding proteins (CaMBPs) [28]. Several experimentally verified CaMBPs are involved in the formation of amyloid-*β* plaques, including amyloid-*β* protein precursor, *β*-secretase, presenilin-1, and ADAM10 [29]. Previous studies have demonstrated that LPL is highly expressed in the pyramidal cells of the hippocampus and involved in the pathogenesis of dementia [30]. Our study has shown that the TBI patients with cognitive deficits had lower serum LPL levels, which is consistent with previous reports that LPL-deficient mice display memory impairments [31]. Moreover, LPL has been suggested to be associated with neurite pathology, and its levels are markedly reduced in the dentate gyrus of AD brains [32]. Thus, its role in post-TBI cognitive impairments is worthy of further investigations.

Additionally, we found that lipid metabolism-associated DEPs, including ApoC-II, Apo(a), cholesteryl ester transfer protein (CETP), and LPL, were significantly altered in the TBI patients with cognitive impairment. It has been suggested that dysregulated lipid metabolism may play an important role in the pathophysiology of post-TBI cognitive impairments. As the main constituent of lipoprotein(a), Apo(a) was validated using ELISA as being significantly upregulated in the TBI patients with cognitive impairment. It has been demonstrated that Apo(a) may be involved in the pathology of dementia by participating in amyloidogenesis and playing a role in neuronal maintenance [33]. Moreover, Apo(a) can alter ApoE isoform metabolism, which suppresses changes related to the development of dementia. ApoC-II and CETP were upregulated in the TBI patients with cognitive impairment. ApoC-II is normally bound to chylomicrons and very-low density lipoproteins and has been found to play an important role in activation of LPL activity [34]. CETP is a key player in lipid metabolism and catalyses the transfer of cholesteryl esters from high-density lipoprotein particles to triglyceride-rich lipoproteins in exchange for triglycerides. In addition, CETP is linked to cerebral cholesterol metabolism and associated with cognitive function [35].

THBS4 and Talin-1 were also both validated using ELISA as being significantly upregulated in the TBI patients with cognitive impairment. In contrast, accumulated data indicate that THBS4 could not only regulate synapse formation but also play an important role in neurite and axon outgrowths [36]. Furthermore, THBS4 has been suggested to play a role in regulating protective astrogenesis from the subventricular zone (SVZ) niche after brain injury in a Notch1-dependent manner [37]. Considering the contrasting results, further investigation on the role of THBS in TBI-induced cognitive impairments is required. Talin-1, which is the key talin member in immune cells, was found to be significantly upregulated in the TBI patients with cognitive impairments as compared to the negative and HC subjects. Previous studies have indicated that Talin-1 plays a role in tumour formation, migration, and metastasis in different types of cancer [38, 39]. However, the impact of Talin-1 on TBI-induced cognitive impairments has yet to be unravelled.

Although the altered serum DEPs were identified and their possible underlying mechanisms were investigated, the present study has several limitations. First, only the serum of TBI patients with cognitive impairments was analysed. To more accurately reflect the pathophysiology of cognitive deficits following TBI, the plasma and CSF from the same individuals with post-TBI cognitive impairments should be analysed using iTRAQ-based quantitative proteomic approaches in the future. Second, the 5 DEPs selected for ELISA validation are not brain specific. Moreover, a correlation between the 5 DEPs and the degree of cognitive impairment has not been established. Third, the present study included only a small number of patients; therefore, additional studies using a larger patient population should be conducted to fully confirm/validate the current findings. Fourth, similar to previous studies [40–42], we also only performed our experiment using one time point, which may result in overinterpretation of the findings. Additional time points would be beneficial for achieving optimal results. Lastly, the aim of this study was to identify the common underlying pathologies of TBI-related cognitive impairments, irrespective of the heterogeneity of the injuries sustained and the variability of the resulting cognitive deficits [43]. Thus, this study included TBI patients with different lesion locations, including those of the frontal lobe, temporal lobe, and parietal lobe, and we did not analytically distinguish these types of TBI patients.

## 5. Conclusion

To the best of our knowledge, the present study was the first to use an iTRAQ-based quantitative proteomic approach to identify DEPs in serum samples obtained from TBI patients with cognitive deficits to better understand the pathophysiology of cognitive impairments following TBI. Using an iTRAQ-based quantitative proteomic analysis, serum proteome alterations in patients with cognitive impairment after TBI were identified, and 56 DEPs were found to be specifically related to TBI-induced cognitive impairment. Moreover, bioinformatic analysis revealed that AD signaling pathways and lipid metabolism are involved in the pathophysiology of cognitive deficits following TBI. However, the limitations of the present study require further investigation and large-scale validation.

## Supplementary Material

Figure S1. Differentially expressed proteins (DEPs) identified in the Alzheimer's disease pathways. The red rectangles indicate the DEPs (CaM ) identified in this study that were significantly increased in the positive group, whereas the green rectangles indicate the DEPs(LPL and GAPD) identified in this study that were significantly decreased in the positive group.

## Figures and Tables

**Table 1:** Demographic characteristics of the enrolled participants.

	Positive	Negative	HC
Number	51	51	51
Female/male	20/31	22/29	24/27
Age	35.7 ± 12.6	36.9 ± 14.8	38.6 ± 11.5
Disease duration (days)	45.10 ± 3.75	49.50 ± 4.79	/
Cause of TBI			
Transport accidents	35	40	/
Fall	10	8	/
Assaults	2	1	/
Others	4	2	/
GCS score at admission median (IQR)	7 (4–9)	8 (4–10)	/
Moderate/severe^①^	31/20	26/25	/
Neurosurgery^②^			
No/yes	28/23	31/20	/
Multiple ICD-10 diagnosis (S06)	43	44	/

Note: IQR, interquartile range. ^①^Moderate/severe indicates the classification of TBI according to GCS score at admission. ^②^Neurosurgery. “Yes” indicates the patients who underwent neurosurgical operative intervention at admission, whereas “No” indicates those who did not.

**Table 2:** Differentially expressed proteins (DEPs) identified using iTRAQ coupled with LC-MS/MS.

Number	Accession	Description	Mascot score	Coverage (%)	MW (kDa)	Fold change		Regulation
						Positive/negative	Positive/HC	
1	A8MT79	Putative zinc-alpha-2-glycoprotein-like 1	26.26	4.90	23	0.767	/	Down
2	O76076	WNT1-inducible-signaling pathway protein 2	72.31	11.60	26.8	0.818	0.768	Down
3	P01598	Ig kappa chain V–I region EU	119.29	26.85	11.8	/	0.827	Down
4	P01616	Ig kappa chain V–II region MIL	83.47	33.04	12	/	1.279	Up
5	P01620	Ig kappa chain V–III region SIE	318.55	59.63	11.8	/	0.824	Down
6	P01625	Ig kappa chain V-IV region Len	150.17	36.84	12.6	/	0.791	Down
7	P01701	Ig lambda chain V–I region NEW	48	15.32	11.4	1.39	/	Up
8	P01717	Ig lambda chain V-IV region Hil	75.53	17.76	11.5	1.319	1.297	Up
9	P01742	Ig heavy chain V–I region EU	58.23	10.26	12.5	1.291	1.449	Up
10	P01764	Ig heavy chain V–III region VH26	109.91	35.04	12.6	/	0.773	Down
11	P01766	Ig heavy chain V–III region BRO	134.83	25.00	13.2	/	0.812	Down
12	P01768	Ig heavy chain V–III region CAM	106.7	23.77	13.7	0.674	0.595	Down
13	P01779	Ig heavy chain V–III region TUR	138	26.72	12.4	0.8	0.749	Down
14	P02655	Apolipoprotein C-II	344.57	57.43	11.3	/	1.353	Up
15	P02751	Fibronectin	4895.14	52.68	262.5	/	0.827	Down
16	P02788	Lactotransferrin	670.88	34.51	78.1	/	1.234	Up
17	P04180	Phosphatidylcholine-sterol acyltransferase	498.47	29.77	49.5	/	0.791	Down
18	P04278	Sex hormone-binding globulin	51.27	5.47	43.8	1.275	1.333	Up
19	P04406	Glyceraldehyde-3-phosphate dehydrogenase	170.08	18.21	36	0.495	0.444	Down
20	P04434	Ig kappa chain V–III region VH (fragment)	65.35	23.28	12.7	0.68	0.781	Down
21	P05155	Plasma protease C1 inhibitor	167.06	13.60	55.1	/	1.208	Up
22	P06753	Tropomyosin alpha-3 chain	123.25	10.88	32.9	/	1.253	Up
23	P06858	Lipoprotein lipase	104.22	7.58	53.1	0.808	/	Down
24	P08185	Corticosteroid-binding globulin	22.27	1.98	45.1	0.801	/	Down
25	P08519	Apolipoprotein(a)	130	25.62	501	1.436	1.62	Up
26	P0C0L5	Complement C4-B	5043.1	65.19	192.6	1.226	/	Up
27	P11021	78 kDa glucose-regulated protein	123.72	10.55	72.3	0.762	/	Down
28	P11597	Cholesteryl ester transfer protein	210.43	12.37	54.7	1.229	/	Up
29	P12814	Alpha-actinin-1	104.07	5.16	103	1.32	1.373	Up
30	P15169	Carboxypeptidase N catalytic chain	69.71	7.21	52.3	0.827	0.808	Down
31	P23142	Fibulin-1	1123.98	42.11	77.2	/	0.833	Down
32	P28066	Proteasome subunit alpha type 5	48.13	4.98	26.4	0.767	/	Down
33	P29122	Proprotein convertase subtilisin/kexin type 6	29.19	1.03	106.4	/	0.777	Down
34	P35443	Thrombospondin-4	226.54	8.84	105.8	1.228	1.405	Up
35	P49913	Cathelicidin antimicrobial peptide	73.93	12.35	19.3	/	0.686	Down
36	P62158	Calmodulin	45.27	26.17	16.8	1.255	1.224	Up
37	P68032	Actin, alpha cardiac muscle 1	244.16	25.20	42	/	1.214	Up
38	P68104	Elongation factor 1-alpha 1	42.01	4.33	50.1	0.77	/	Down
39	P68366	Tubulin alpha-4A chain	66.67	6.25	49.9	/	1.362	Up
40	P68871	Hemoglobin subunit beta	196.67	52.38	16	/	1.303	Up
41	Q02985	Complement factor H-related protein 3	160.25	11.52	37.3	/	1.212	Up
42	Q14515	SPARC-like protein 1	219.59	17.77	75.2	/	0.753	Down
43	Q6ZV73	FYVE, RhoGEF, and PH domain-containing protein 6	36.67	1.12	160.7	1.29	/	Up
44	Q86UX7	Fermitin family homolog 3	22.07	2.40	75.9	0.8	0.798	Down
45	Q8IWZ6	Bardet-Biedl syndrome 7 protein	39.01	1.12	80.3	/	1.24	Up
46	Q92540	Protein SMG7	34.49	0.70	127.2	/	1.222	Up
47	Q92954	Proteoglycan 4	233.65	7.05	151	/	0.829	Down
48	Q96KK5	Histone H2A type 1-H	82.29	21.88	13.9	/	0.735	Down
49	Q9H4B7	Tubulin beta-1 chain	57.95	6.21	50.3	0.74	0.81	Down
50	Q9H6R4	Nucleolar protein 6	33.17	0.61	127.5	0.793	/	Down
51	Q9NUD7	Uncharacterized protein C20orf96	27.39	2.48	42.8	/	1.229	Up
52	Q9UBU7	Protein DBF4 homolog A	42.74	0.89	76.8	/	1.367	Up
53	Q9UHG3	Prenylcysteine oxidase 1	248.97	15.84	56.6	/	0.819	Down
54	Q9UK55	Protein Z-dependent protease inhibitor	615.93	31.98	50.7	0.825	0.717	Down
55	Q9Y490	Talin-1	61	1.61	269.6	1.248	1.427	Up
56	Q9Y5C1	Angiopoietin-related protein 3	40.67	1.74	53.6	/	1.268	Up

Note: regulation: up or down indicates the DEPs that were upregulated or downregulated, respectively, in the positive group relative to the reference groups.

**Table 3:** KEGG pathways associated with the differentially expressed proteins (DEPs) identified by iTRAQ analysis.

Number	Pathway ID	Pathway	DEPs with pathway annotation	Mapped DEPs	*P* value
1	ko05322	Systemic lupus erythematosus	3	P0C0L5, P12814, Q96KK5	0.001338
2	ko05133	Pertussis	3	P05155, P0C0L5, P62158	0.003080
3	ko04510	Focal adhesion	4	P02751, P12814, P35443, Q9Y490	0.010071
4	ko05144	Malaria	2	P35443, P68871	0.011312
5	ko05010	Alzheimer's disease	3	P04406, P06858, P62158	0.015594
6	ko05130	Pathogenic *Escherichia coli* infection	2	P68366, Q9H4B7	0.021948
7	ko04260	Cardiac muscle contraction	2	P06753, P68032	0.026523
8	ko04261	Adrenergic signaling in cardiomyocytes	3	P06753, P62158, P68032	0.027736
9	ko04970	Salivary secretion	2	P49913, P62158	0.032064
10	ko04610	Complement and coagulation cascades	2	P05155, P0C0L5	0.032890
11	ko04512	ECM-receptor interaction	2	P02751, P35443	0.037802
12	ko05410	Hypertrophic cardiomyopathy (HCM)	2	P06753, P68032	0.038242
13	ko05414	Dilated cardiomyopathy (DCM)	2	P06753, P68032	0.039573
14	ko04145	Phagosome	3	P35443, P68366, Q9H4B7	0.041505
15	ko05146	Amoebiasis	2	P02751, P12814	0.043672
16	ko04540	Gap junction	2	P68366, Q9H4B7	0.045073

## References

[1] Morawska M. M., Büchele F., Moreira C. G., Imbach L. L., Noain D., Baumann C. R. (2016). Sleep modulation alleviates axonal damage and cognitive decline after rodent traumatic brain injury. *Journal of Neuroscience*.

[2] Rabinowitz A. R., Levin H. S. (2014). Cognitive sequelae of traumatic brain injury. *Psychiatric Clinics of North America*.

[3] Cristofori I., Levin H. S. (2015). Traumatic brain injury and cognition. *Handbook of Clinical Neurology*.

[4] Walker K. R., Tesco G. (2013). Molecular mechanisms of cognitive dysfunction following traumatic brain injury. *Frontiers in Aging Neuroscience*.

[5] Titus D. J., Wilson N. M., Freund J. E. (2016). Chronic cognitive dysfunction after traumatic brain injury is improved with a phosphodiesterase 4B inhibitor. *Journal of Neuroscience*.

[6] Wang X., Li A., Guo Y. (2016). iTRAQ-based proteomics screen identifies LIPOCALIN-2 (LCN-2) as a potential biomarker for colonic lateral-spreading tumors. *Scientific Reports*.

[7] Li M., Peng F., Li G. (2016). Proteomic analysis of stromal proteins in different stages of colorectal cancer establishes Tenascin-C as a stromal biomarker for colorectal cancer metastasis. *Oncotarget*.

[8] Muenchhoff J., Poljak A., Song F. (2015). Plasma protein profiling of mild cognitive impairment and Alzheimer's disease across two independent cohorts. *Journal of Alzheimer's Disease*.

[9] Sharma R., Gowda H., Chavan S. (2015). Proteomic signature of endothelial dysfunction identified in the serum of acute ischemic stroke patients by the iTRAQ-based LC-MS approach. *Journal of Proteome Research*.

[10] Zhang P., Zhu S., Li Y. (2016). Quantitative proteomics analysis to identify diffuse axonal injury biomarkers in rats using iTRAQ coupled LC-MS/MS. *Journal of Proteomics*.

[11] Wu P., Zhao Y., Haidacher S. J. (2013). Detection of structural and metabolic changes in traumatically injured hippocampus by quantitative differential proteomics. *Journal of Neurotrauma*.

[12] Crawford F., Crynen G., Reed J. (2012). Identification of plasma biomarkers of TBI outcome using proteomic approaches in an APOE mouse model. *Journal of Neurotrauma*.

[13] Yi L., Shi S., Wang Y. (2016). Serum metabolic profiling reveals altered metabolic pathways in patients with post-traumatic cognitive impairments. *Scientific Reports*.

[14] Norup A., Kristensen K. S., Siert L., Poulsen I., Mortensen E. L. (2011). Neuropsychological support to relatives of patients with severe traumatic brain injury in the sub-acute phase. *Neuropsychological Rehabilitation*.

[15] Gouvier W. D., Blanton P. D., LaPorte K. K., Nepomuceno C. (1987). Reliability and validity of the Disability Rating Scale and the Levels of Cognitive Functioning Scale in monitoring recovery from severe head injury. *Archives of Physical Medicine and Rehabilitation*.

[16] White N. M. A., Masui O., DeSouza L. V. (2014). Quantitative proteomic analysis reveals potential diagnostic markers and pathways involved in pathogenesis of renal cell carcinoma. *Oncotarget*.

[17] Huang W., Liang Q., Chen J. (2015). Quantitative proteomic analysis of synovial tissue from rats with collagen-induced arthritis. *RSC Advances*.

[18] Xu D.-D., Deng D.-F., Li X. (2014). Discovery and identification of serum potential biomarkers for pulmonary tuberculosis using iTRAQ-coupled two-dimensional LC-MS/MS. *Proteomics*.

[19] Wang C., Wei L., Shi L. (2015). Screening and identification of five serum proteins as novel potential biomarkers for cured pulmonary tuberculosis. *Scientific Reports*.

[20] Mi H., Poudel S., Muruganujan A., Casagrande J. T., Thomas P. D. (2016). PANTHER version 10: expanded protein families and functions, and analysis tools. *Nucleic Acids Research*.

[21] Szklarczyk D., Franceschini A., Wyder S. (2015). STRING v10: protein-protein interaction networks, integrated over the tree of life. *Nucleic Acids Research*.

[22] Xing Z., Xia Z., Peng W. (2016). Xuefu Zhuyu decoction, a traditional Chinese medicine, provides neuroprotection in a rat model of traumatic brain injury via an anti-inflammatory pathway. *Scientific Reports*.

[23] Sivanandam T. M., Thakur M. K. (2012). Traumatic brain injury: a risk factor for Alzheimer's disease. *Neuroscience and Biobehavioral Reviews*.

[24] Plassman B. L., Grafman J. (2015). Traumatic brain injury and late-life dementia. *Handbook of Clinical Neurology*.

[25] El Kadmiri N., Slassi I., El Moutawakil B. (2014). Glyceraldehyde-3-phosphate dehydrogenase (GAPDH) and Alzheimer's disease. *Pathologie Biologie*.

[26] Hara M. R., Snyder S. H. (2006). Nitric oxide-GAPDH-Siah: a novel cell death cascade. *Cellular and Molecular Neurobiology*.

[27] Huang J., Xiong N., Chen C. (2011). Glyceraldehyde-3-phosphate dehydrogenase: activity inhibition and protein overexpression in rotenone models for Parkinson's disease. *Neuroscience*.

[28] O'Day D. H., Myre M. A. (2004). Calmodulin-binding domains in Alzheimer's disease proteins: extending the calcium hypothesis. *Biochemical and Biophysical Research Communications*.

[29] O'Day D. H., Eshak K., Myre M. A. (2015). Calmodulin binding proteins and Alzheimer's disease. *Journal of Alzheimer's Disease*.

[30] Yang H., Zhou T., Wang H. (2015). Lipoprotein lipase deficiency leads to *α*-synuclein aggregation and ubiquitin C-terminal hydrolase L1 reduction. *Neuroscience*.

[31] Xian X., Liu T., Yu J. (2009). Presynaptic defects underlying impaired learning and memory function in lipoprotein Lipase-deficient mice. *The Journal of Neuroscience*.

[32] Gong H., Dong W., Rostad S. W. (2013). Lipoprotein lipase (LPL) is associated with neurite pathology and its levels are markedly reduced in the dentate gyrus of Alzheimer's disease brains. *Journal of Histochemistry and Cytochemistry*.

[33] Kunutsor S. K., Khan H., Nyyssönen K., Laukkanen J. A. (2016). Is lipoprotein (a) protective of dementia?. *European Journal of Epidemiology*.

[34] Amar M. J. A., Sakurai T., Sakurai-Ikuta A. (2015). A novel apolipoprotein C-II mimetic peptide that activates lipoprotein lipase and decreases serum triglycerides in apolipoprotein E-knockout mice. *Journal of Pharmacology and Experimental Therapeutics*.

[35] Arias-Vásquez A., Isaacs A., Aulchenko Y. S. (2007). The cholesteryl ester transfer protein (CETP) gene and the risk of Alzheimer's disease. *Neurogenetics*.

[36] Yang H. J., Ma S. P., Ju F. (2016). Thrombospondin-4 promotes neuronal differentiation of NG2 cells via the ERK/MAPK pathway. *Journal of Molecular Neuroscience*.

[37] Benner E. J., Luciano D., Jo R. (2013). Protective astrogenesis from the SVZ niche after injury is controlled by Notch modulator Thbs4. *Nature*.

[38] Xu N., Chen H.-J., Chen S.-H. (2016). Upregulation of Talin-1 expression associates with advanced pathological features and predicts lymph node metastases and biochemical recurrence of prostate cancer. *Medicine*.

[39] Xu Y.-F., Ren X.-Y., Li Y.-Q. (2015). High expression of Talin-1 is associated with poor prognosis in patients with nasopharyngeal carcinoma. *BMC Cancer*.

[40] Zhang X., Yin X., Yu H. (2012). Quantitative proteomic analysis of serum proteins in patients with Parkinson's disease using an isobaric tag for relative and absolute quantification labeling, two-dimensional liquid chromatography, and tandem mass spectrometry. *Analyst*.

[41] Zhan Y., Yang Y.-T., You H.-M. (2014). Plasma-based proteomics reveals lipid metabolic and immunoregulatory dysregulation in post-stroke depression. *European Psychiatry*.

[42] Wang Q., Su X., Jiang X. (2016). iTRAQ technology-based identification of human peripheral serum proteins associated with depression. *Neuroscience*.

[43] Jenkins P. O., Mehta M. A., Sharp D. J. (2016). Catecholamines and cognition after traumatic brain injury. *Brain*.

