# Supplementary material for: Serum Proteome Alterations in Patients with Cognitive Impairment after Traumatic Brain Injury Revealed by iTRAQ-Based Quantitative Proteomics
Source: Biomed Res Int. 2017 Jan 30;2017:8572509. doi: 10.1155/2017/8572509 (PMC5303854; doi:10.1155/2017/8572509)
Supplement: Supplementary file 1 — Figure S1. Differentially expressed proteins (DEPs) identified in the Alzheimer's disease pathways. The red rectangles indicate the DEPs (CaM ) identified in this study that were significantly increased in the positive group, whereas the green rectangles indicate the DEPs(LPL and GAPD) identified in this study that were significantly decreased in the positive group. [file 8572509.f1.docx]

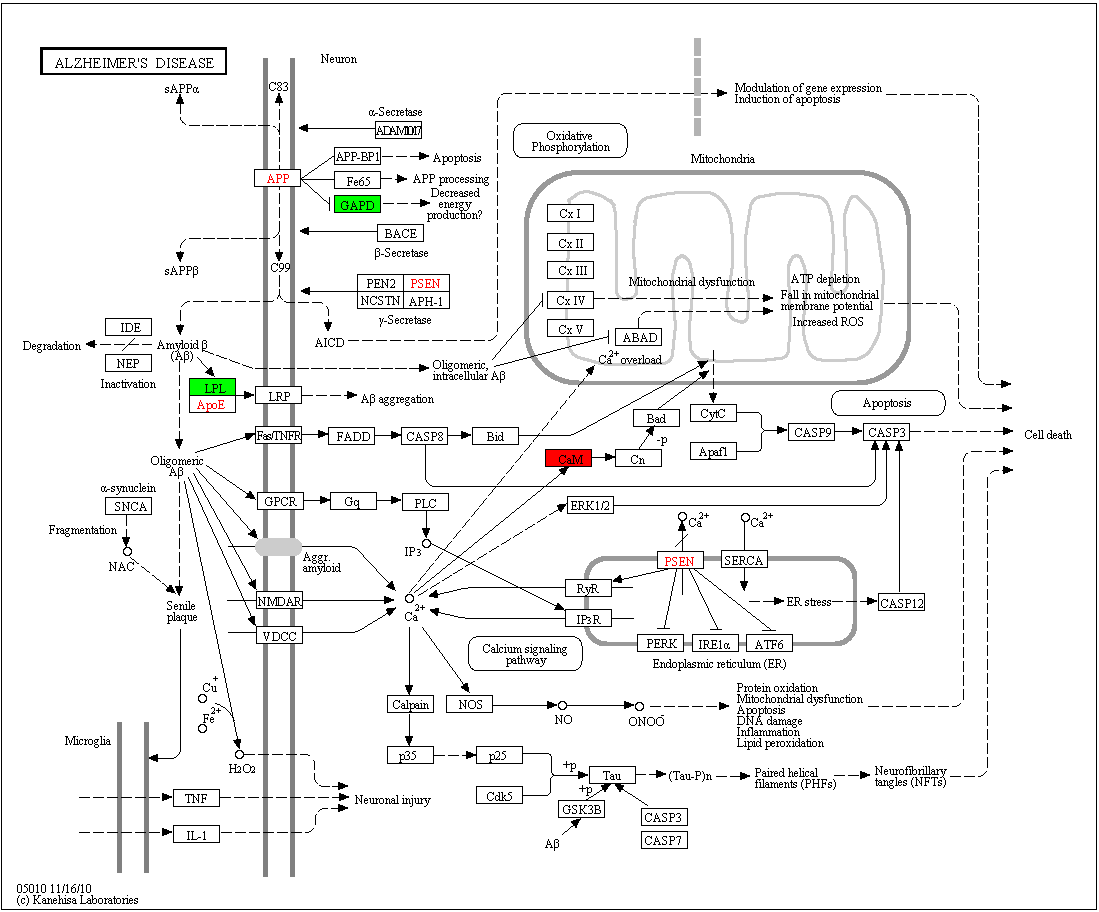


Figure S1. Differentially expressed proteins (DEPs) identified in the Alzheimer's disease pathways. The red rectangles indicate the DEPs identified in this study that were significantly increased in the positive group, whereas the green rectangles indicate the DEPs identified in this study that were significantly decreased in the positive group.
